# ‘A good fit?’ Bringing the sociology of footwear to the clinical encounter in podiatry services: a narrative review

**DOI:** 10.1186/s13047-018-0253-6

**Published:** 2018-03-20

**Authors:** Emily Nicholls, Victoria Robinson, Lisa Farndon, Wesley Vernon

**Affiliations:** 1 Sociology, University of Portsmouth, Portsmouth, UK; 2 Centre for Women’s Studies, University of York, York, UK; 3 Podiatry Services, Sheffield Teaching Hospitals NHS Foundation Trust, Sheffield, UK

**Keywords:** Footwear, Podiatry, Shoes, Sociology, Qualitative research, Methodology, Identity

## Abstract

**Background:**

This narrative review explores the ways in which drawing on theories and methods used in sociological work on footwear and identity can contribute to healthcare research with podiatrists and their patients, highlighting recent research in this field, implications for practice and potential areas for future development.

Traditionally, research within Podiatry Services has tended to adopt a quantitative, positivist focus, developing separately from a growing body of sociological work exploring the importance of shoes in constructing identity and self-image. Bringing qualitative research drawing on sociological theory and methods to the clinical encounter has real potential to increase our understanding of patient values, motivations and – crucially – any barriers to adopting ‘healthier’ footwear that they may encounter. Such work can help practitioners to understand why patients may resist making changes to their footwear practices, and help us to devise new ways for practitioners to explore and ultimately break down individual barriers to change (including their own preconceptions as practitioners). This, in turn, may lead to long-term, sustainable changes to footwear practices and improvements in foot health for those with complex health conditions and the wider population.

**Conclusion:**

A recognition of the complex links between shoes and identity is opening up space for discussion of patient resistance to footwear changes, and paving the way for future research in this field beyond the temporary ‘moment’ of the clinical encounter.

## Background

With research suggesting that over 60% of women and 30% of men suffer from pain when wearing shoes [1], there is a clear need for further research into the numerous factors affecting an individual’s footwear choices, particularly when such choices might be assessed as ‘poor’ by practitioners. Research shows there is a correlation between ill-fitting shoes and specific foot pain [2]; wearing ‘fashion’ shoes such as high heels or slip-on pumps is associated with a number of issues from blisters and bunions to toe deformities, hallux valgus and ulcers [3–5]. In addition, up to 10% of the UK population experience ‘disabling’ foot pain [6], which may of course be exacerbated by certain footwear choices. At the same time, the sale of fashion shoes has soared over recent years [7] and podiatrists report ongoing challenges in encouraging people to adopt ‘healthier’ footwear [8]. High heels have been associated with the development of foot pathologies including clawed toes, bunions and foot pain [3] and more recently this type of footwear has been linked to a number of different foot injuries presenting at one emergency department; the most commonly affected areas were the ankle, foot and toes [9].

This of course raises a number of questions; why might people continue to wear shoes that cause discomfort and even pain? Why do some patients disregard professional advice about changing their footwear, despite an awareness that certain shoes may exacerbate existing conditions, limit mobility and even increase the risk of further complications? And finally, how can podiatrists better develop a dialogue with patients to support them in making realistic, sustainable changes to their footwear choices? Podiatrists and patients are frequently seen as at opposite ends of a spectrum; ‘healthy’ footwear may be regarded by patients as unfashionable and unwearable despite the benefits of changing their shoes in line with professional advice and recommendations such as the ‘Healthy Footwear guide’ [8]. This article explores the ways in which a turn to recognising and exploring shoe choice from a more *sociological* perspective can help us to understand and explore some of the reasons why patients may resist making changes to their footwear, whilst illuminating the importance of shoes to identity and sense of self. It begins by outlining some of the links between identity and footwear made in the field of sociology, before highlighting examples of healthcare research in the last decade that has started to adopt qualitative methodologies and recognise and explore the centrality of shoes to patients’ sense of identity. The article then explores the implications for practice of this growing body of research, and makes recommendations for future research, particularly further research beyond the clinical encounter.

The material highlighted in this narrative review presents an overview of a number of journal articles and previous studies reviewed initially in 2015 as part of the literature review for a project on footwear and identity. A second ‘sweep’ of relevant literature was undertaken in 2017 to identify any more recent developments in this field. Papers were sourced through Google Scholar and through searching specific targeted health journals using keywords such as ‘podiatry’, ‘identity’ and ‘concordance’. Whilst 80 articles were reviewed as part of this process, only the most relevant for elucidating the synergies between footwear, identity and sociological studies are included here.

## Shoes, self and sociology

Embodied and everyday footwear practices have been a relatively neglected area of research in both sociological and health contexts [10]. However, shoes are not ‘neutral’ objects, in fact they are likely to be ‘the single most expensive item in people’s outfit’ [10]. Any attempts to understand the relationship between patients and their shoes in healthcare contexts would benefit from acknowledging previous sociological research that highlights the central role footwear can play in presentation and management of the self, identity and the body.

Previous sociological research has noted that shoes are important throughout the life course, during key life transitions [11, 12], and also in the more ‘everyday’ production of identity [13]. Identity can be theorised here as fluid, embodied and constructed in relation to others. In other words, our identities are not stable and fixed, rather, we are always in a process of ‘becoming’ through how we present ourselves and interact with the world [14]. The production of identities can be understood as a fluid process dependent on contextual, temporal and other factors; for example, Dilley et al. [15] explore the importance of ‘occasions’ and ‘non-occasions’ in shaping women’s everyday choices regarding footwear and assessing when to wear high heels. For sociologists, footwear can be, among other things, a mundane and everyday way of presenting the self, a means for transition or a ‘vehicle’ or ‘passport’ which can provide insights into who we are or want to be with extraordinary implications for transforming identities [11, 13, 15].

Whilst sociological insights into shoes, identity and transition have been hugely useful, there has traditionally remained something of a separation between sociological perspectives and research in healthcare settings. Much previous sociological research in this field has utilised a more qualitative, in-depth approach and tended to focus on the general population [11, 13, 15], rather than those with specific foot conditions. In contrast, healthcare research on patient shoe choice has traditionally taken a more positivist perspective and tended to use quantitative methods [16]. Such research has also focused largely on practitioner perspectives [17], meaning the voices and experiences of patients may remain silenced or excluded. However, there are some notable exceptions to these trends, and a growing body of research is bringing more qualitative, social-science perspectives and methodologies to clinical contexts or settings.

## Widening understandings of ‘fit’: social science in healthcare contexts

Over the past decade or so, a number of key studies in healthcare settings have utilised methods, theories and approaches drawing on the social sciences to provide a richer and more in-depth insight into patients’ relationships with their feet and shoes.

Williams et al. [17] carried out qualitative research interviews with patients with Rheumatoid Arthritis (RA) to explore their experiences of orthopaedic footwear. In a recognition of the links between shoes, identity and femininity, they found that female patients often felt that their sense of feeling feminine or ‘sexy’ was being eroded when they were required to abandon their fashion shoes. For several participants, orthopaedic or specialist footwear also compounded an existing negative self-image and could be linked with feelings of shame, anger and humiliation. Those who did wear their prescribed footwear frequently suggested that it could limit their engagement with social activities, which is important because those with chronic conditions may be at higher risk of social isolation more generally. This highlights the practical consequences that changing shoes can have on patients’ embodied, everyday lives.

Building on this work, Goodacre and Candy [18] have elicited further in-depth discussions around footwear with patients with RA. An excellent example of work which adopts a holistic approach to footwear and really considers the wider effects of clothes and shoes on bodies and self-presentation, the researchers undertook semi-structured and in-depth interviews with 15 women with RA in the North-West of England to explore body image and clothing choices. Whilst mobility was a priority, there was often a sense of compromise apparent in shoe choice, as ‘comfortable’ shoes that aided mobility might also be described as ‘clumpy’ and undesirable, and might limit clothing choices and participants’ ability to present themselves as they wanted. Crucially, there was also a sense of the feet transforming from something that could be adorned and displayed to something to be kept hidden. The powerful feelings of shame, stigma and exclusion that could be associated for women with wearing the ‘wrong’ shoes may go some way towards explaining why over 70% of the footwear choices of women with RA could be classified as ‘poor’ [19]. Such findings suggest that the ‘right’ shoes must be more than just a good physical fit, but ideally should ‘fit’ the wearer mentally too. Seferin and Van der Linden [20] extend this idea theoretically by differentiating between physical comfort and psychological comfort (e.g. feeling attractive or feeling shoes ‘fit’ the occasion rather than just ‘fit’ physically). Comfort is thus not necessarily measured objectively or rationally, but is rather a complex and subjective way of assessing shoes, meaning that physical comfort is not always the top priority in footwear selection.

Whilst such contributions are hugely important, there has been a tendency for research to focus on older populations [2, 21, 22] or those with health conditions such as diabetes, gout or RA [17–19, 23, 24], and studies from a more sociological, qualitative perspective still remain comparatively rare. However, Branthwaite et al. [25] offer a fresh perspective in their research with teenage girls regarding their shoe purchases over 6 months. For the young women involved in the research, fashion was prioritised over physical fit, function or health considerations. Once again, the links between shoe choice, identity and body image were highlighted. This work with younger populations is important because research suggests that shoe choice in youth and midlife can have a huge impact on foot health in later life. Furthermore, front-line foot health practitioners have an important preventative role to play in educating the wider public *before* specific conditions and complications arise [26].

More recently, research with patients and podiatrists explores why patients may resist practitioner advice around changing their footwear practices and draws more explicitly on social science theories and methodologies, bringing together a research team of podiatrists and social scientists [27]. Building directly on a previous sociological research project in this area [11] which received a high level of interest from podiatrists, semi-structured interviews and focus groups were utilised alongside more innovative methods such as photography and shoe diaries in order to explore patients’ relationships with shoes and real or perceived barriers to adopting ‘healthier’ footwear. Findings confirmed and extended earlier work suggesting that ‘fit’ is more than just physical, widening out understandings of fit to consider the mental and social fit of a pair of shoes, and whether they are ‘fit for purpose’ and suit the patients’ lifestyle, hobbies, self-image and the ways in which they wish to present themselves.

It is clear that adopting qualitative, social science methods has facilitated a more in-depth exploration of the values, motivations and preferences of patients, and the challenges or constraints they may encounter when advised to make changes to their footwear. In particular, prior research has contributed to a widening of our understanding of what constitutes a ‘good fit’ in terms of footwear. Whilst physical fit and comfort are likely to be important considerations for a number of patients, if shoes do not ‘fit’ a person more widely, they are likely to either be disregarded or abandoned over the longer term, or to give rise to feelings of shame, resentment and emotional or psychological discomfort.

## Implications for practice

In order to ensure that changes to footwear are positive, sustainable and long-term, practitioners need to consider ‘fit’ in a more holistic sense, and explore patient values, preferences and any potential resistance to change. Resistance has traditionally been regarded as a negative behaviour to be overcome; however, more recent research suggests that engaging with and identifying patient resistance can help practitioners to expose – and start to break down – individual barriers to change [28, 29].

An important step for research in this field is to continue to bridge the gap between sociological research and clinical practice. Whilst the increasing recognition of the links between footwear and identity is of course welcome, it is essential that the findings of such work are fed back into podiatrist training and practice that recognises that patient values, motivations and practices in relation to the embodied experiences of choosing and wearing shoes are complex. Recent research in this field [27] has led to the development of an online toolkit, recommendations for practice and a visual tool, all designed to directly inform practice in patient consultations and to encourage practitioners to reconceptualise fit more broadly and find ways to discuss this wider understanding of fit with their patients. Although there is further research to be done to consider how developments such as the toolkit can more directly inform training and practice, the findings of a range of previous research suggest that tools such as Motivational Interviewing (MI) are likely to be of value in clinical settings [30].

It is important to recognise the obstacles that over-stretched practitioners may experience to introducing meaningful dialogue through MI into clinical encounters, particularly in terms of the limited time available in each patient consultation [31]. Yet even small changes in the ways in which practitioners communicate with patients can have an influence on rates of acceptance and adherence to treatment or lifestyle changes [32]. MI is a consultation strategy designed to facilitate an in-depth discussion of patient values and motivations in order to address and seek to resolve any ambivalences towards behaviour change [30]. Within a time limited consultation, elements of what Gabbay et al. [30] call ‘brief MI’ - open-ended questions, reflective listening and summarising – can be used with impact to discuss and address patient obstacles to making long-term footwear changes.

## Future directions and emerging research

Research in healthcare settings that draws on social science theories, approaches and methodologies can provide in-depth insights into patient values and highlight the complex relationships that patients may have with their shoes and feet. Whilst the use of semi-structured interviews is becoming more established in this field, future research could make further use of innovative methods (such as photography, videos, shoe diaries, shoe shopping trips with patients) which have until recently been relatively underutilised in sociological research on health and illness [33]. Effective links must also be made between research and ‘real world’ clinical encounters to allow the research findings to be fed back into professional practice (for example through podiatrist training or the design of practical tools for use in consultation).

Future research would benefit from looking more specifically at how elements of ‘brief MI’ might be adopted in practice to help achieve behavioural change, and from continuing to look *beyond* the clinical encounter at the wider role of shoes in people’s everyday, embodied lives. In wider research around patient compliance to treatment, conversation analysis has frequently been used to explore micro-level interaction within clinical encounters [29, 34]. However, the clinical encounter is merely a ‘moment’ and healthcare research moving forward can usefully draw theoretically on sociological approaches such as symbolic interactionism to explore lived experiences; i.e. what people *do* after their appointment, rather than what they might *say* they will do during a clinical encounter. This is important when there may be discrepancies in what patients commit to in clinical encounters and what they actually do in their everyday lives outside the patient-practitioner interaction. Symbolic interaction encourages a focus on people’s *everyday* embodied practices beyond the medical context and how such practices construct particular aged, classed and gendered subjects [35]. Furthermore, despite the prevalence of foot pain in the UK [5], only 4% of the UK population are currently accessing Podiatry Services. Research that goes beyond analysing interaction in the clinical setting and explores the centrality of shoes to both the wider public as well as those receiving treatment has the potential to enhance the preventative role of Podiatry Services and improve public education [26].

With the current climate of NHS cost-cutting and austerity, and increasing shifts towards self-care and managing conditions through everyday lifestyle changes under a ‘neoliberalised’ healthcare system [36], future research in this area will continue to be important. Research suggests that Podiatry patients can struggle with self-care [8], yet in a climate of cuts and cost-saving measures, it is likely that the onus will increasingly rest on patients to manage their own conditions. Therefore, research exploring the motivations behind patient experiences and barriers to change they encounter is timely and welcomed. The consequences of poor shoe choices can be significant, and increasing opportunities for genuine, individualised dialogue between practitioners and patients may help those patients to successfully make ‘healthier’ shoe choices that still align with their values and preferences. This, in turn, may lead to improved long-term foot health and ultimately reduce the burden placed on the NHS [37].

## Conclusion

To conclude, it is clear that adopting a more sociological perspective to healthcare research on shoes and footwear can add an important new dimension to our understanding of patient behaviour within or outside the clinical encounter. Specifically, exploring patient values, motivations and preferences in relation to shoe choice can help to shed light on some of the barriers to change that patients may encounter when advised to adopt ‘healthier’ footwear. An understanding of the centrality of shoes to people’s sense of self and identity may help practitioners to recognise that changing footwear practices is not a simple, rational or neutral process. Rather, shoes may be intimately bound up with a patient’s sense of who they are – or who they want to be – meaning change may not come easily.

The increasing use of more qualitative methods – particularly innovative methods such as shoe diaries and videos – offers further opportunities for practitioners to really explore patient values and priorities and their relationship with their feet and footwear. Moving forward, further use of in-depth methods that are embedded into patients’ everyday lives and explore their daily practices and habits will provide real scope to understand the ways in which practitioner advice is adopted, resisted or ignored (and of course why this is so) outside and beyond the clinical encounter.
